# Spectrum of *ERCC6*-Related Cockayne Syndrome (Type B): From Mild to Severe Forms

**DOI:** 10.3390/genes15040508

**Published:** 2024-04-18

**Authors:** Jacopo Sartorelli, Lorena Travaglini, Marina Macchiaiolo, Giacomo Garone, Michaela Veronika Gonfiantini, Davide Vecchio, Lorenzo Sinibaldi, Flaminia Frascarelli, Viola Ceccatelli, Sara Petrillo, Fiorella Piemonte, Gabriele Piccolo, Antonio Novelli, Daniela Longo, Stefano Pro, Adele D’Amico, Enrico Silvio Bertini, Francesco Nicita

**Affiliations:** 1 Unit of Neuromuscular and Neurodegenerative Disease, Bambino Gesù Children’s Hospital, IRCCS, P.zza Sant’Onofrio 4, 00165 Rome, Italy; 2 Laboratory of Medical Genetics, Translational Cytogenomics Research Unit, Bambino Gesù Children’s Hospital, IRCCS, P.zza Sant’Onofrio 4, 00165 Rome, Italy; 3 Rare Diseases and Medical Genetics Unit, Bambino Gesù Children’s Hospital, IRCCS, 00165 Rome, Italy; 4 Neurology, Epilepsy and Movement Disorder Unit, Bambino Gesù Children’s Hospital, IRCCS, P.zza Sant’Onofrio 4, 00165 Rome, Italy; 5 Department of Neuroscience, Mental Health and Sensory Organs, Faculty of Medicine and Psychology, Sapienza University of Rome, 00185 Rome, Italy; 6 Rehabilitation Unit, Bambino Gesù Children’s Hospital, IRCCS, P.zza Sant’Onofrio 4, 00165 Rome, Italy; 7 Neuroradiology Unit, Imaging Department, Bambino Gesù Children’s Hospital, P.zza Sant’Onofrio 4, 00165 Rome, Italy; 8 Developmental Neurology Unit, Bambino Gesù Children’s Hospital, IRCCS, P.zza Sant’Onofrio 4, 00165 Rome, Italy

**Keywords:** ataxia, cerebellar hypoplasia, hypomyelination, leukodystrophy, genotype–phenotype, neurofilament light chain

## Abstract

(1) Background: Cockayne syndrome (CS) is an ultra-rare multisystem disorder, classically subdivided into three forms and characterized by a clinical spectrum without a clear genotype-phenotype correlation for both the two causative genes *ERCC6* (CS type B) and *ERCC8* (CS type A). We assessed this, presenting a series of patients with genetically confirmed CSB. (2) Materials and Methods: We retrospectively collected demographic, clinical, genetic, neuroimaging, and serum neurofilament light-chain (sNFL) data about CSB patients; diagnostic and severity scores were also determined. (3) Results: Data of eight *ERCC6/*CSB patients are presented. Four patients had CS I, three patients CS II, and one patient CS III. Various degrees of ataxia and spasticity were cardinal neurologic features, with variably combined systemic characteristics. Mean age at diagnosis was lower in the type II form, in which classic CS signs were more evident. Interestingly, sNFL determination appeared to reflect clinical classification. Two novel premature stop codon and one novel missense variants were identified. All CS I subjects harbored the p.Arg735Ter variant; the milder CS III subject carried the p.Leu764Ser missense change. (4) Conclusion: Our work confirms clinical variability also in the *ERCC6*/CSB type, where manifestations may range from severe involvement with prenatal or neonatal onset to normal psychomotor development followed by progressive ataxia. We propose, for the first time in CS, sNFL as a useful peripheral biomarker, with increased levels compared to currently available reference values and with the potential ability to reflect disease severity.

## 1. Introduction

Cockayne syndrome (CS) is a multisystem disorder with a range of possible manifestations, including postnatal growth failure and progressive microcephaly with neurodegeneration as remarkable features, and is classified into three forms based on psychomotor development and age of symptoms onset. CS type I, or classic CS, is characterized by normal prenatal growth with growth failure and developmental abnormalities appearing in the first two years of life with delayed independent walking, and minimal verbal communication but good peer interaction. CS type II, the severe infantile form, shows growth failure and eye anomalies at birth, with little or no postnatal neurologic development. CS type III has almost normal psychomotor development and onset of progressive symptoms after the age of two years old. Finally, cerebro-oculo-facio-skeletal (COFS) syndrome is considered the most severe end of the spectrum, with prenatal onset of some manifestations [1,2,3,4,5,6].

Recessive pathogenic variants in *ERCC6* or *ERCC8*, two genes necessary for the transcription-coupled nucleotide excision repair (TC-NER) pathway, a subtype of NER involved in repairing ultraviolet-induced DNA damage in actively transcribed genes, are responsible for most CS cases. A small percentage of subjects with overlapping phenotypes harbors pathogenic variants in other genes (i.e., de novo *MORC2* variants; *ERCC2* and *ERCC3* variants in the xeroderma pigmentosum–CS overlap complex) [2]. Weak genotype–phenotype correlations are reported for both *ERCC6*- (CS type B) and *ERCC8*-related (CS type A) cases [1,2,7,8,9].

In this study, we present eight subjects with CS caused by *ERCC6*/CSB variants, underlining its wide spectrum and discussing a possible genotype–phenotype correlation. 

## 2. Materials and Methods

This single-center, observational, retrospective study was carried out at the IRCCS Bambino Gesù Children’s Hospital, Rome, Italy. 

Main inclusion criteria were a clinical diagnosis of one of the three CS forms, confirmed by the presence of homozygous or compound heterozygous variants in one of the CS-related genes validated through segregation analysis with parents’ gene testing. 

Exclusion criteria were the absence of genetic confirmation, as mentioned above, or the absence of minimal dataset information regarding clinical (e.g., neurodevelopmental, neurological exam, dermatological, and ophthalmological) data; Nfl measurement and neuroimaging and neurophysiology data were considered not obligatory for inclusion in the study. 

We retrospectively collected demographic, clinical, genetic data, and, when available, other (i.e., neuroimaging, neurophysiologic, and laboratory) data of recruited patients. 

Single gene sequencing, targeted next-generation sequencing (i.e., targeted panels or clinical exome), and/or array comparative genomic hybridization were variably performed. Variants were classified according to ACMG criteria.

We determined CS diagnostic and severity scores, as previously described by Spitz et al. [7]. Diagnostic scores include ten (clinical) or twelve (clinical–neuroradiological) criteria, which have a different rating, and their sum generates the final score. According to this score, the CS diagnosis can be considered with high, moderate, or low probability. Similarly, a severity score can be calculated, considering the degree of involvement in 5 clinical and developmental aspects, with a higher score in milder forms.

NfL concentrations were measured using the Human Simple Plex assay kit (ProteinSimple) on an Ella device (ProteinSimple), according to the manufacturer's instructions. The calibration of Ella was performed using the in-cartridge factory standard curve, and plasma samples were measured with a 1:2 dilution in Sample Diluent provided by the kit. Triplicates were automatically performed in the Simple Plex assay micro-fluidic platform, and data were expressed as pmol/mL.

Parents or guardians of all participants provided written informed consent for genetic testing and for participation in research studies.

## 3. Results

We enrolled eight patients (three females and five males) with a genetically confirmed diagnosis of CS type B, caused by pathogenic variants in *ERCC6*. Patients’ demographic and genetic features are detailed in Table 1. 

Patients mean age at enrolment was 11 years old (range: 2.5–17 years), with a mean age at diagnosis of 5 years old (range: 2.5–17 years). From a clinical point of view, four patients had clinical signs consistent with a CS I form, three patients with a CS II, and one patient with CS III. Systemic and neurologic features with CS scores are detailed in Table 2 and Table 3, respectively.

**Figure 1:**
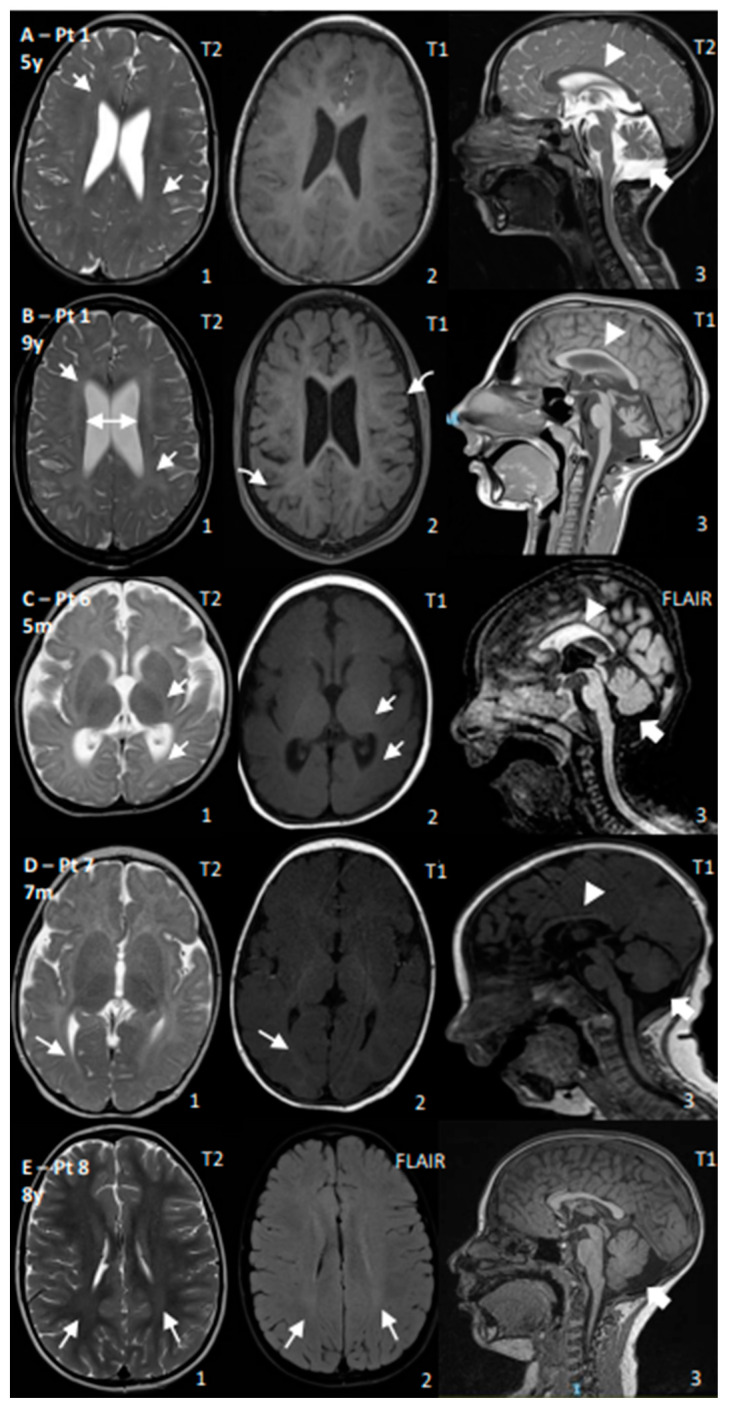
Representative brain MRI findings of cerebral and cerebellar involvement in CS subjects. (**A**–**E**): Brain MRI of patients 1 (lines **A**,**B**), 6 (line **C**), 7 (line **D**), and 8 (line **E**). In patient 1 with CS type I (lines **A**,**B**), thin corpus callosum (arrowheads in **A3**,**B3**), progressive cerebellar (thick arrows in **A3**,**B3**) and cerebral (curve arrows in **B2**) atrophy with lateral ventricle enlargement (double arrow in **B1**), and worsening of hypomyelination of sovratentorial white matter (thin arrows in **A1**,**B1**) are observed; hypomyelination has a patchy distribution, especially at onset, and involves both subcortical and deep white matter. In patients with CS type II (patients 6 (line **C**) and 7 (line **D**)), early reductions in brain and cerebellar volume (thick arrows in **C3**,**D3**) are present, together with a thin corpus callosum (arrowheads in **C3**,**D3**) and reductions in total amount of white matter and myelin deposit compared to age (thin arrows in **C1**,**C2**,**D1**,**D2** indicate reduced myelination of posterior limb of the internal capsulae and optic radiation). In patient with CS type III (patient 8, line **E**), a mild hyperintense signal of the posterior white matter (thin arrows in **E1**,**E2**) and increase in posterior fossa with normal cerebellum (thick arrow in **E3**) are evident.

CS I patients (n. 1–4) developed progressive spastic-ataxic syndrome after an initial normal or mildly delayed motor development with a poor language. Patients reached an ability to walk independently or with support and subsequently showed a degree of regression. One patient had a pure spastic phenotype and spasticity was the main complaint, needing medical treatment. Three cases required surgical tendon elongation. Microcephaly was a common feature, together with photosensitivity, growth failure (associated also to dysphagia in three patients), and hypertransaminasemia, although this last feature was mild and only temporarily detected in the first years of life in two cases and subsequently normalized during follow up. Brain MRIs (Figure 1A,B) evidenced progressive cerebral and cerebellar volume losses (i.e., atrophy) and evidence of white matter involvement featuring hypomyelination in all the three subjects. Typical ophthalmologic alterations characterized by initial signs of lens opacity and retinal pigmentation were present in one case. Clinical diagnostic scores were 7–9 out of 20 and clinical–radiological 13–19 out of 39. Severity scores ranged from 3 to 8 out of 15. 

CS II patients (n. 5–7) presented tetraparesis, with minimal motor development during the first year of life, and severe intellectual disability with absent language. Brain MRI (Figure 1C,D) and neurophysiologic studies were performed on two out of three subjects: early involvement with cerebral, cerebellar, and white matter volume reduction as well as reduced myelination for age and abnormal visual evoked potentials were detected. Brainstem auditory evoked potentials showed a pontine anomaly in one case; peripheral demyelinating sensory motor neuropathy was detected in one case. Spasticity was addressed pharmacologically in two patients, and one patient presented an isolated episode of afebrile seizure with occipital interictal epileptic discharges on the EEG, which did not require antiseizure medications. A bilateral congenital cataract was present in one subject, while two presented enophthalmia and skin photosensitivity. Growth failure—starting in utero for two patients—and hypertransaminasemia were observed in all three patients. Diagnostic clinical scores ranged from 8 to 10 (out of 20) and, where neuroimaging was available, they reached 13 and 16 (out of 39), respectively (see Table 2). They had the lowest severity scores of the whole cohort, scoring 1 and 2 (out of 15). 

A single CS III patient (n. 8) presented with normal gross motor and language development, mild ataxia from early childhood, and mild white matter hyperintensity on T2/FLAIR images at brain MRI (Figure 1E). The next-generation sequencing panel targeted for ataxia genes evidenced *ERCC6* variants, in the absence of other systemic signs suggestive of CS, as shown by the low diagnostic scores (clinical 0/20; clinical–radiological 3/39) and high severity score (14/15). 

Genetic diagnosis was performed through single gene testing for clinical suspicion of CS in two patients (n. 3 and 4), clinical exome sequencing for clinical suspicion of CS in four patients (n. 2, 5, 6, and 7), while in two cases (n. 1 and 8), it was obtained by targeted next-generation sequencing (NGS) panel for inherited ataxias or white matter disorders. Chromosome 10 deletions, including part or full *ERCC6*, were detected in two cases (n. 2 and n. 4) by array comparative genomic hybridization in compound heterozygosity with another variant previously detected by NGS. Parental testing was available for all patients, revealing that all parents were heterozygous carriers. A total of nine pathogenic variants, including one missense, one intronic, and seven frameshift or premature stop codon variants were detected in the reported subjects. Also, two chromosome 10 deletions involving the *ERCC6* gene were found. All patients carried at least one variant leading to a premature stop codon. Three novel variants (e.g., p.Leu764Ser, p.Arg928fsTer5, and p.Tyr627Ter) were disclosed. The missense p.Leu764Ser was a novel variant classified as a likely pathogenic (class IV) according to the ACMG criteria, while the remaining variants were classified as pathogenic (class V). The p.Arg735Ter variant recurred in homozygous or compound heterozygous states in all the four CSI subjects, while the missense p.Leu764Ser was present in the mildest CS III subject.

The analysis of serum neurofilament light chain (sNFL) was available in 5 out of 8 cases: increased values were detected in all cases, ranging from 45 to 270 pg/mL with the highest levels in the two younger and more severe CS II (n. 5 and 7) subjects and mildest levels in one CSI (n. 1) and CSIII (n. 8) subject. 

## 4. Discussion

We present a series of *ERCC6*/CSB subjects with clinical and genomic variabilities. 

Possible manifestations of CS include progressive microcephaly and growth failure, visual impairment (due to corneal opacification, cataracts, and retinal dystrophy), enophthalmia, neurodevelopmental delay evolving into intellectual disability and spastic-ataxia, abnormal neuroimaging, with both white and gray matter involvement, peripheral demyelinating neuropathy, sensorineural hearing loss, cutaneous photosensitivity, dental anomalies, and hepatic, kidney, or vascular dysfunction. Among these, postnatal growth failure and progressive microcephaly with neurodegeneration appear to be striking features, which strongly suggest CS, particularly within CSII but also in the CSI form [1,2,5]. Therefore, adapted growth centiles were developed to help clinicians to better assess the patients’ height, weight, and head circumference, and to determine their nutritional status. This aspect is central to CS, since, together with the genetic background, dysphagia is also a recurrent symptom, both contributing to the reduced target for growth [10].

Each feature of CS may have a spectrum of severity; a negative prognostic value has been attributed to age of cataract onset and to more prominent skin photosensitivity [11]. Diagnostic clinical criteria of CS were initially introduced by Nance and Berry in 1992 and, subsequently, diagnostic scores with both clinical and radiologic features were proposed in 2021 [6,7]. These include ten (clinical) or twelve (clinical–neuroradiological) criteria, respectively, selected as the most statistically discriminant between a CS and a non-CS cohort, which generate high, moderate, and low probability thresholds for CS diagnosis. Applying them retrospectively to our series at the time of achievement of genetic diagnosis, we found three out of eight patients with a “high likelihood” of CS, and three out of eight with a “low likelihood” of CS, underlying the difficulty of reaching a clinical–radiological diagnosis without genetic testing in this syndrome due to the broad phenotypic variability. The severity score, which is calculated determining the level of manifestations in five clinical and developmental aspects, on the other hand, appeared to correlate well with the classic clinical classification, with higher scores in milder forms [11]. Patients presented in our study showed different degrees of involvement at their current ages, ranging from severe in utero growth restriction to mild isolated ataxia. Common features were microcephaly, growth failure, poor language development, hypertransaminasemia (even if mild and transient in two CS I subjects), and photosensitivity. Eye involvement was less frequent than reported in the literature. From a neurologic point of view, ataxia and spasticity appeared to be cardinal findings, with different degrees of severity. The most severe form (i.e., CS II) partially overlapped with COFS syndrome. Interestingly, age at diagnosis was lower for CS II (1 year old for all subjects) than CS I patients (mean: 7.3 years old; range: 3–17 years old). This may be related to the younger age of symptoms/signs onset in CS II than CS I, but also to the striking appearance of cardinal features in CS II that correlates with higher diagnostic scores. CS I often presented less prominent cardinal features, thus giving rise to intermediate diagnostic scores and a possible overlap with other conditions (e.g., congenital ataxia and leukodystrophy). Additionally, it may be challenging to clinically suspect the milder CS III form. It is worth noting that new genetic techniques are allowing the early diagnosis and phenotypic expansion of several diseases, especially in cases with unusual characteristics and/or milder phenotypes as observed in the CS III subject. Some reports described diagnoses of CS III with initial isolated developmental delay or ataxic–dystonic signs or mild isolated ataxia followed by neurodegeneration in adulthood [12,13,14,15,16,17]. The abovementioned data seem to indicate that the *ERCC6-*related CS represents a spectrum ranging from very severe to milder forms in which the clinical characterization remains important from a diagnostic (i.e., easier clinical recognition of severe form vs. more difficult clinical recognition of milder forms) and prognostic point of view, since earlier CS II forms appear to have the most severe involvement and the worse outcome, while the outcome for milder CS III forms remains unclear, given the limited number of reported patients and poor long-term follow-up data. Natural history studies will clarify these aspects. 

Neuroimaging features for the reported patients seem to reflect clinical severity, with early signs of cerebral, cerebellar, and white matter volume reduction/loss in severe CS II, progressive cerebro-cerebellar atrophy and hypomyelination in CS I, and only mild white matter involvement in the only CS III subject. Permanent hypomyelination, with some patchy and progressive white matter involvement, appeared to be more easily identifiable in CS II subjects, although it is not easy to recognize it as primary or secondary hypomyelination. Brain calcifications are also reported later in the CS literature, appearing months to years after the onset of neurologic symptoms in the putaminal areas of CS I patients and in putaminal and cortical sulci areas of CS II patients [18]. We did not observe striking signs of calcifications, although they may have been underestimated since no computed tomography scan or brain MRI sequences specific for calcium deposit (e.g., susceptibility-weighted images) were available. CS should be considered in the differential diagnosis of patients presenting with various degrees of ataxia and/or spasticity and MRI findings of white matter (i.e., hypomyelination) and cerebro-cerebellar involvement (i.e., progressive atrophy) also in the absence of calcifications. 

From a genetic perspective, all our patients carried mutations in *ERCC6*, a gene central to TC-NER. In fact, it assists RNA polymerases with transcription blocks caused by DNA damage-recruiting nucleotide excision repair proteins to remove alterations [19,20]. In addition, it has been shown to work as a transcription factor in mitochondrial DNA metabolism [21]. Recessive *ERCC6* variants account for up to 70–75% of CS patients [1] and they often lead to premature stop codons, while missense mutations are less common, as confirmed by our cohort, where all patients carried at least one stop codon mutation and only one patient (n. 8) out of eight harbored a missense variant. Interestingly, CS I subjects in our study presented the same p.Arg735Ter mutation and the milder CS III subject the novel p.Leu764Ser missense mutation. In addition, partial chromosome 10 deletions were detected in our cohort, as previously described in the literature [22].

Neurofilament light chain, the smallest subunit of the cytoskeletal protein neurofilaments, has been proposed as a clinically relevant biomarker of neuronal axonal injury and degeneration in several disorders, such as Charcot–Marie–Tooth disease, Metachromatic Leukodistrophy, or Hereditary Spastic Paraplegias [23,24,25]. To the best of our knowledge, its use has not been described in CS patients. Interestingly, sNFL levels in CS subjects appeared to be above the 99th percentile, according to recently published reference values [26], and, thus, it could represent a useful peripheral biomarker. Our findings suggest a possible correlation between sNFL level and disease severity that needs to be assessed in larger studies, together with the influence of other possible variables (a trend toward age has been described for the control subjects) and of possible changes during the natural history of the disease. This could be linked to CS pathophysiology, which is characterized by progressive neuronal degeneration, as shown in a CS animal model (*csb*-1 mutant *Caenorhabditis elegans*), which leads both to brain atrophy with white and grey matter loss with NFL release and elevations in cerebrospinal fluid and serum [27,28]. 

This work has several limitations, mostly represented by its retrospective nature and the absence of a standardized evaluation for every patient; additionally, the small number of subjects does not allow a statistical analysis for correlations and the generalization of our findings. 

## 5. Conclusions

The CS concept is evolving from a syndrome with specific major features to a clinical spectrum. This clinical variability is evident also in the *ERCC6*/CSB type, where manifestations may range from severe involvement with prenatal or neonatal onsets up to normal psychomotor development followed by progressive ataxia. CS II should be considered in subjects with a striking appearance of cardinal features, while classic CS I should be considered in patients showing various degrees of ataxia and spasticity and brain MRI findings featuring hypomyelination and cerebro-cerebellar involvement (i.e., progressive atrophy) with or without calcifications. Finally, milder CS III forms are probably under-recognized and their descriptions may be useful for improving early clinical recognition, the genotype–phenotype correlation, and the identification of prognostic factors. Increased sNFL level could be a useful tool both for the recognition of the neurodegenerative process and as a biomarker of clinical severity, but its validity must be assessed in larger studies. 

## Figures and Tables

**Table 1:** Demographic and genetic features.

Patient	CS Type	Age (Years)	Sex	Age at Diagnosis (Years)	Pathogenic Variants	Genetic Classification
1	CS I	17	F	17	*ERCC6*: homozygous c.2203C>T; p.Arg735Ter	CSB
2	CS I	7	F	3	*ERCC6*: c.2203C>T, p.Arg735Ter; 10q11.22-q11.23 microdeletion	CSB
3	CS I	16	M	4.5	*ERCC6*: c. 2203C>T, p.Arg735Ter; c.2096_2097insC, p.Leu700ValfsTer60	CSB
4	CS I	21	M	5	*ERCC6*: c.2751C>T, p.Gly917Gly paternally inherited; c.2203C>T, p.Arg735Ter paternally inherited; chromosome 10 5.5Mb deletion maternally inherited	CSB
5	CS II	2.5	M	1	*ERCC6*: c.2784_2785delAG, p.Arg 928fsTer5; c.1881C>A, p.Tyr627Ter	CSB
6	CS II	5	F	1	*ERCC6*: homozygous c.1431_1432delGA, p.K478TfsTer9	CSB
7	CS II	3	M	1	*ERCC6*: c.2383-1G>T, splice acceptor variant; c.3259C>T, p.Arg1087Ter	CSB
8	CS III	9	M	9	*ERCC6*: c.2096dupC, p.Leu700fsTer60; c.2291T>C, p.Leu764Ser	CSB

**Table 2:** Systemic features.

Patient CS Type	Ophtalmologic	Gestation and Birth	Photosensitivity	Growth Failure	Dysphagia	Hypertransaminasemia	Other Features	Diagnostic Score: CS; CRS	Severity Score
1 CS I	Normal vision	Normal	Yes	Yes, <−5 SD	No	Yes (at age 1 y), then normalized	-	9/20; 19/39	5/15
2 CS I	Small opacity in left eye lens; reduced retinic pigmentation (salt and pepper appearance)	Reduced head circumference growth during gestation	Yes	Yes, −2 SD	Yes	Yes (at age 4 y), then normalized	-	8/20; 16/39	8/15
3 CS I	Astigmatism	CSec at 37 GW due to fetal bradycardia	Yes	Yes, <−5 SD	Yes	Yes	Photophobia. Cryptorchdism.	7/20; 13/39	4/15
4 CS I	Hypermetropy	Normal	Yes	Yes, <−5 SD	Yes	Yes	Scoliosis. Cryptorchidism.	7/20; N/A	3/15
5 CS II	Normal vision	Reduced growth during last month	Yes	Yes, <−5 SD	No	Yes	Enophtalmia.	10/20; N/A	2/15
6 CS II	Bilateral congenital cataract, with recurrence after surgical treatment	CSec at 36 GW due to growth restriction; SGA at birth	Yes	Yes, <−5 SD	Yes	Yes	-	10/20; 16/39	1/15
7 CS II	Hypermetropy	Right clubfoot and bilateral pyelectasis	No	Yes, <−5 SD	Yes	Yes	Thoracic kiphosis. Cryptorchidism. Enophtalmia.	8/20; 13/39	2/15
8 CS III	Normal vision	Normal	No	No	No	No	Monolateral kidney dysplasia.	0/20; 3/39	14/15

Legend: CS, Clinical Score; CRS, Clinical Radiologic Score; CSec, Caesarian Section; GW, Gestational Week; N/A, Not Available.

**Table 3:** Neurologic features.

Pat.	CS Type	Spasticity	Ataxia	Seizures	Microcephaly	Maximum Developmental Milestone (Age)	Language Development	sNFL (pg/mL)	Brain MRI Findings	Neurophisiologic Findings
1	CS I	Yes	Yes	No	Yes, −4 SD	Walk (2 y, lost 3 y)	Poor	45	Age: 4 y and 9 y: Progressive cerebral and cerebellar atrophy; thin CC; permanent hypomyelination	ERG: normal; VEP: increased latency; BAEP: increased I–V latency; SSEP: increased CCT
2	CS I	Yes	Yes	No	Yes, −3 SD	Walk with support (2 y)	Poor	99–136	Age: 10 m and 1.5 y: Progressive cerebral and cerebellar atrophy; thin CC; WM reduction and hypomyelination	NCV: normal
3	CS I	Yes	Yes	No	Yes, <−5 SD	Walk with support (1.7 y)	Poor	N/A	Age: 4 y: Permanent hypomyelination	ERG: reduced amplitude
4	CS I	Yes	No	No	Yes, <−5 SD	Walk (1 y), then regression	Poor	N/A	N/A	N/A
5	CS II	Yes	No	No	Yes, <−5 SD	Head control (4.5 m)	Poor	175	N/A	N/A
6	CS II	Yes	Yes	Yes	Yes, <−5 SD	No acquisition	No acquisition	N/A	Age: 5 m: Cerebral, cerebellar, CC, and WM reductions; reduced myelination for age	EEG: occipital anomalies; VEP: poor cortical definition; BAEP: increased I–V latency
7	CS II	Yes	Yes	No	Yes, <−5 SD	Head control (5 m)	Poor, then regression	198–270	Age 7 m: Cerebral, cerebellar, CC, and WM reductions; reduced myelination for age	NCV: demyelinating SM neuropathy; ERG: normal; VEP: high latency
8	CS III	No	Yes	No	No	Walk (1 y)	Normal	49	Age: 8 y: mild posterior WM T2 hyperintensity; increased posterior fossa	BAEPs: increased I–V latency; ERG/VEP: normal; NCS: mild decrease in motor conduction velocity in the lower limbs

Legend: Pat., patient; y, years; m, months; n.v., normal value; BAEP, brainstem auditory evoked potential; CC, corpus callosum; CCT, central conduction time; EEG, eletroencephalogram; ERG, electroretinoram; N/A, not available; NFL, neurofilament light chain (expressed as range when several values were available); NCV, nerve conduction velocity; SSEP, somato-sensory evoked potentials; SM, sensory motor; VEPs, visual evoked potentials; WM, white matter.

## Data Availability

The original contributions presented in the study are included in the article; further inquiries can be directed to the corresponding authors.
